# The Long Noncoding RNA Cytoskeleton Regulator RNA (CYTOR)/miRNA-24-3p Axis Facilitates Nasopharyngeal Carcinoma Progression by Modulating GAD1 Expression

**DOI:** 10.1155/2023/6027860

**Published:** 2023-02-13

**Authors:** Jingwei Du, Wangbo Yu, Lijuan Peng, Tao Zhang

**Affiliations:** ^1^Department of Otolaryngology-Head and Neck Surgery, The First Affiliated Hospital of Jinan University, Guangzhou 510630, China; ^2^Department of Otolaryngology-Head and Neck Surgery, Nanchong Central Hospital, The Second Clinical Medical College, North Sichuan Medical College, Nanchong 637000, Sichuan, China; ^3^Department of Otolaryngology-Head and Neck Surgery, Affiliated Hospital of North Sichuan Medical College, Nanchong 637000, Sichuan, China; ^4^Department of Pathogen Biology, School of Basic Medical Sciences and Forensic Medicine, North Sichuan Medical College, Nanchong 637000, Sichuan, China

## Abstract

Nasopharyngeal carcinoma (NPC) is a head and neck epithelial carcinoma that is unusually prevalent in Southeast Asia. Noncoding RNAs, including lncRNA and miRNA, and their target genes are considered vital regulators of tumorigenesis and the progression of NPC. However, the detailed underlying mechanisms of GAD1 involved in the regulation of NPC need to be further elucidated. In the present study, we identified that GAD1 was significantly upregulated in NPC tissues. GAD1 overexpression is promoted, while genetic knockdown of GAD1 suppresses proliferation, colony formation, migration, and invasion of NPC cells. Bioinformatics analysis and a luciferase reporter assay demonstrated that GAD1 is a direct target gene of miR-24-3p. In NPC tissues, miR-24-3p was downregulated and the lncRNA CYTOR was upregulated. CYTOR was sponged to suppress the function of miR-24-3p. CYTOR regulates GAD1 expression via modulating miR-24-3p. The CYTOR/miR-24-3p/GAD1 axis is converged to modulate the growth, migration, and invasion of NPC cells. In conclusion, the study identified a novel axis for the regulation of NPC cell growth, providing new insights into the understanding of NPC.

## 1. Introduction

Nasopharyngeal carcinoma (NPC), a carcinoma originating from the nasopharyngeal mucosa epithelium, is one of the most severe head and neck tumors [1, 2]. The global incidence of NPC is over 120,000 cases annually, with over 70% of the cases occurring in Southeast Asia, especially southern China [3]. There are three primary pathogenic factors for NPC, including Epstein–Barr virus (EBV) infection, genetic susceptibility, and environmental factors [4]. Early diagnosis of NPC is generally not possible, and radical surgical resection is prohibitive due to the anatomical structure of the nasopharyngeal mucosa and its proximity to vital organs [5, 6]. Because most pathological types of NPC are nonkeratinizing undifferentiated carcinomas, which are highly malignant and prone to cervical lymph node metastasis, 20% of patients develop local-regional recurrence or distant metastasis due to tumor radio-resistance or chemotherapy resistance, resulting in treatment failure [2, 7]. Delineating the molecular mechanisms of NPC recurrence and metastasis is imperative for the identification of potential targets to increase the sensitivity of the NPC to treatment and improve its prognosis.

Glutamate decarboxylase 1 (GAD1) is a rate-limiting enzyme of glutamate and gamma-aminobutyric acid (GABA) [8, 9]. GAD1 and GABA are important neural regulators in adult mammals, but studies have found that GAD1 and GABA are also present in many nonneural tissues. For example, GAD1 expression is significantly upregulated in many tumor tissues, such as rectal cancer, breast cancer, lymphoma, and gastric cancer [10, 11]. GAD1 is significantly overexpressed in patients with metastatic prostate cancer, suggesting its potential application as a prostate-specific biomarker [12, 13]. As a target of the *β*-catenin/TCF pathway, GAD1 is highly expressed in Wilms' tumors [14]. In addition, GAD1 expression is significantly elevated in microsatellite unstable (MSI) colon cancer compared to microsatellite stable (MSS) cancer [15]. Recent studies have demonstrated that GAD1 and GABA regulate tumor cell proliferation, especially that of stem cells [16]. However, the role of GAD1 and the detailed regulatory mechanisms of NPC remain incompletely understood.

LncRNA and miRNA are small noncoding RNAs that play critical roles in regulating the development and metastasis of NPC [17, 18]. miR-24-3p is downregulated in NPC and targets Jab1/CSN5 to modulate NPC cell function [19]. miR-24-3p-enriched exosomes impede T-cell function by targeting GFG11, which is a potential biomarker for NPC [20]. Furthermore, the lncRNA cytoskeleton regulator RNA (CYTOR) is elevated in NPC tissues and cells and promotes NPC development via miR-163-targeted ANXA2 [21]. However, the potential regulation of GAD1 by miR-24-3p or CYTOR has not been explored.

## 2. Materials and Methods

### 2.1. Bioinformatic Analysis of NPC Dataset

To identify the key genes that play critical roles in NPC, we reanalyzed the NPC dataset published in GEO (GSE12452), which contains 10 nontumor nasopharyngeal epithelial tissue samples and 31 NPC tissue samples [22–24]; and GSE64634, which contains four normal nasopharyngeal tissue samples and 12 nasopharyngeal carcinoma specimens [25]. Briefly, raw CEL files from the Affymetrix Human Genome U133 Plus 2.0 microarray platform were downloaded and imported, along with the 32 GSM samples, into R (version 3.6). Gene expression levels were then analyzed, and differentially expressed genes were identified using the limma package [26]. Data were then plotted in a volcano plot with *P* < 0.01 and a log2-transformed expression fold change >1.0 for further evaluation.

### 2.2. Cell Culture and Transfection

Human NPC cell lines (CNE-1 and 5-8F) were purchased from the American Type Culture Collection (ATCC, Manassas, VA, USA). The cells were cultured in Dulbecco's modified Eagle medium (DMEM; Thermo Scientific, Waltham, MA), containing 10% fetal bovine serum (FBS; Gibco, Grand Island, MA, USA), 0.5% penicillin, and 0.5% streptomycin (Gibco), and cultured in a humidified incubator under 5% CO_2_ at 37°C. The GAD1 and CYTOR genes were extracted and cloned into the pEGFP-C1 vector (Addgen), and siRNA fragments against GAD1 and miR-24-3p mimics and inhibitors were purchased from GenePharma (Shanghai, China). For transfection, the media was changed to DMEM without FBS, and the cells were then transfected with Lipofectamine 2000 reagent (Invitrogen, Grand Island, NY, USA) for 48 h, with the exception to the MTT assays, which were performed at 24 h, 48 h, and 72 h.

### 2.3. Immunohistochemistry

We collected 36 pairs of adjacent tumor and NPC tumor tissues from 36 patients who received surgical treatment in the Nanchong Central Hospital from Jan 2007 to Dec 2009. Written consents were signed by the patients, and the study protocol was approved by the ethics committee of Nanchong Central Hospital in accordance with the Declaration of Helsinki. For immunohistochemistry analysis, the samples embedded in paraffin were cut into sections of 5 *β*M. The following procedures were conducted as reported [27, 28]. Briefly, sections were dewaxed and heat-treated to retrieve epitopes by immersing in a citrate buffer solution at pH 6.0 in an autoclave at 121°C for 1 min. Then, the sections were washed and incubated with 3% hydrogen peroxide for 1 h and blocked with normal goat serum for 1 h. For immunostaining with GAD1, the sections were incubated with anti-GAD1 antibody (1 : 1000, Abcam, Cambridge, MA, USA) overnight at 4°C. Sections with no primary antibody were used as the negative control. Then, sections were incubated with an HRP-labeled secondary antibody for 1 h at room temperature. Then, the sections were visualized with microscopy (BX51, Olympus, Germany). For the GAD1 score in IHC staining, the percentage of neoplastic cells with clear staining were used for quantification: 0, no staining; 1, less than 30% of cells stained in scattered individual cells; 2, less than 50% of cells stained; 3, 50–80% of cells stained; and 4, greater than 80% of cells stained.

### 2.4. Quantitative RT-PCR (qRT-PCR)

For qRT-PCR [29], the total RNA of clinical or cell samples was reverse-transcribed with EasyScript cDNA Synthesis SuperMix, and qRT-PCR was performed with SYBR Premix Ex Taq™ II (TaKaRa, Japan), according to the manufacturer's protocol. Primer sequences are presented in Table 1. Gene expression levels were normalized to U6 or actin.

### 2.5. Western Blot Analysis

The procedure was performed as reported [30]. Total protein cell lysates were isolated with RIPA buffer (Beyotime, Shanghai, China) on ice and centrifuged for 15 min at 14000 rpm. Thirty microliters of total protein from each sample were subjected to SDS-PAGE. The separated proteins were then transferred to PVDF membranes. Subsequently, the membranes were sealed with 5% skim milk for 1 h, and after washing, the membranes were incubated with primary antibodies against GAD1 (#ab228710, 1 : 1000; Abcam, MA, USA) or Actin (#ab8227, Abcam) overnight at 4°C. After washing three times with TBST, membranes were incubated with HRP-conjugated secondary antibodies at room temperature for 1 h. After washing, an ECL kit (Beyotime, cat. no. #P0018S) was used to label the protein bands, and band intensity was quantified using ImageJ and normalized to control.

### 2.6. MTT Assay

For the MTT assay, transfected cells were seeded on a 96-well plate (1 × 10^4^ cells/well) and then incubated with the MTT kit (#C0009 M; Beyotime) for 4 h at 1 day, 2 days, and 3 days. Prior to harvest, 100 *µ*l Formazan solution was added to each well and incubated for an additional 4 h. The absorbance of each well was measured at 570 nm. The values were normalized to those of day 0 control.

### 2.7. Colony Formation Assay

For the colony formation assay, 500 transfected cells/well were plated on a 6-well plate and cultured for 7 days. Colonies were then stained with 0.5% crystal violet (Beyotime) and fixed with 4% paraformaldehyde (Beyotime) for colony quantification.

### 2.8. Transwell Assay for Migration and Invasion

Transfected or untransfected cells were collected and replanted in the upper chamber of a Transwell system (Corning, MA, USA). The chamber was coated with Matrigel (BD Biosciences) for the invasion assay but not the migration assay. The lower chamber contained a full culture medium. After 24 h, migrated or invaded cells were stained with 0.5% crystal violet. Images were then taken, and cell numbers were counted.

### 2.9. Dual-Luciferase Reporter Assay

The sequence of wild-type GAD1 or the CYTOR 3′UTR with a potential recognition site targeting miR-24-3p was synthesized and cloned into luciferase reporter vectors (Promega, Madison, WI, USA), as was a mutant 3′UTR. NPC cells were then transfected with miR-24-3p mimic or the indicated plasmids and cotransfected with Renilla luciferase plasmids as an internal control. After 48 h transfection, cells were harvested and luciferase activity was measured using the Dual-Luciferase Reporter System (Promega), according to the manufacturer's instructions.

### 2.10. Statistical Analysis

All data were presented as the mean ± SD from at least three independent experiments. The statistical analysis was conducted with GraphPad Prism. A student's *t*-test was used for pairwise comparison between two groups, and an ANOVA was used for comparison between multiple groups. *P* < 0.05 was considered statistically significant.

## 3. Results

### 3.1. GAD1 is Upregulated in Nasopharyngeal Cancer Tissues

**Figure 1:**
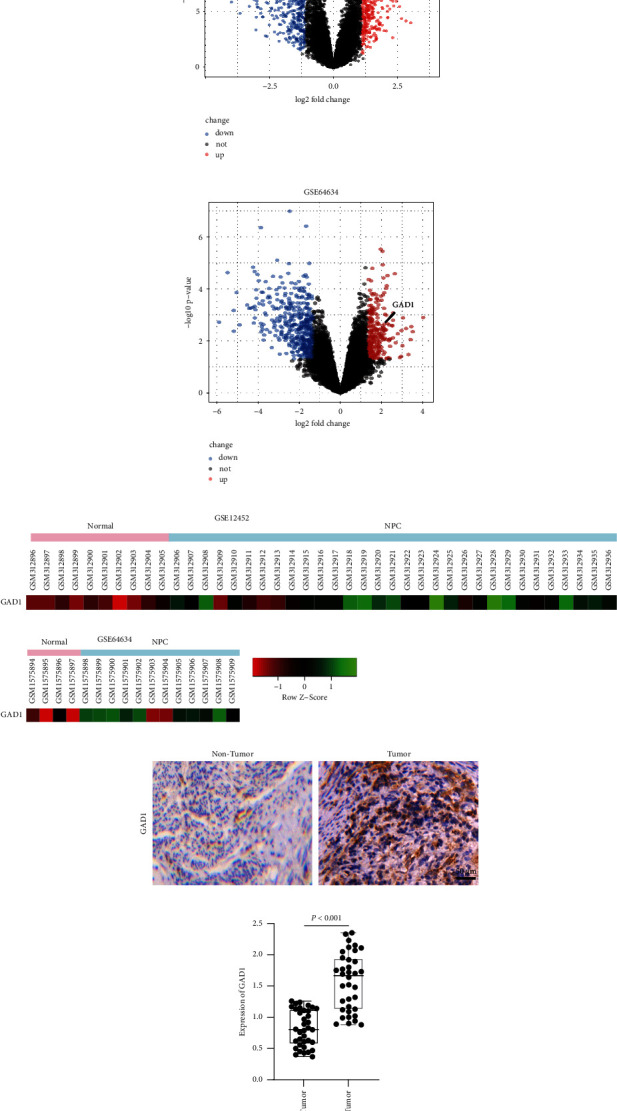
GAD1 is upregulated in NPC tissues. The differentiated expressed genes in public datasets of GSE12452 and GSE64634. The volcanos are shown in (a, b), the normalized expression levels of GAD1 in the two datasets are shown in (c), the expression of GAD1 in our collected samples of NPC is shown in (d) by histochemistry, and the statistical data are shown in (e).

To explore the role of GAD1 in NPC, we first reanalyzed the transcriptome datasets GSE12452 and GSE64634 published in GEO. Differentially expressed genes were analyzed (Figures 1(a) and 1(b)). GAD1 expression was upregulated in both datasets. Scaled expression between the two datasets is represented as a heatmap (Figure 1(c)). These data indicate that GAD1 is upregulated in nasopharyngeal cancer tissues.

### 3.2. GAD1 is Critical for the Growth, Migration, and Invasion of Nasopharyngeal Cancer Cells

**Figure 2:**
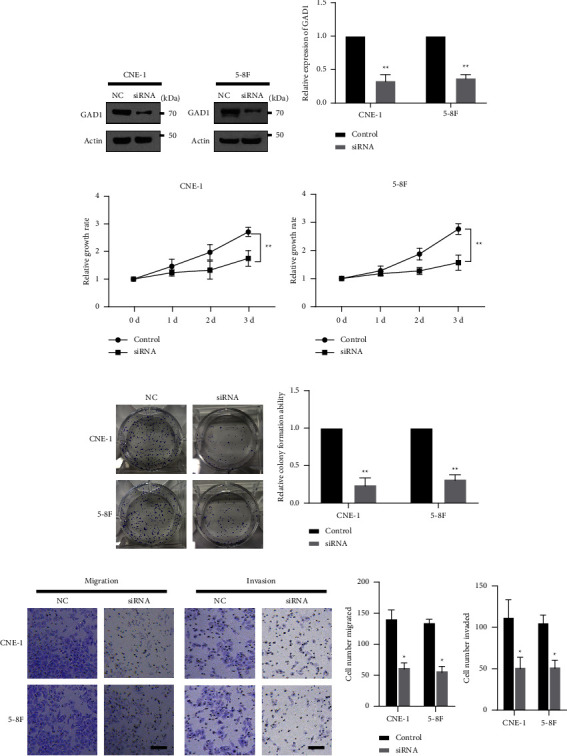
GAD1 is necessary for the development of NPC cells. (a) siRNA fragments against GAD1 were synthesized and transfected into two NPC cell lines, CNE-1 and 5-8F. Then, the lysates were subjected for western blotting for the detection of GAD1. The statistical data are shown in (b). NPC cells were transfected with siRNA fragments against GAD1 for 24 h, and then the cells were subjected to the MTT assay to detect the cell growth rate (c, d), to colony formation assay (e, f), and to the Transwell assay to detect cell migration and invasion (g, h). Scale bar, 20 *β*m; ^*∗*^ denotes *P* < 0.05; ^*∗∗*^ denotes*P* < 0.01; ^*∗∗∗*^ denotes *P* < 0.001.

**Figure 3:**
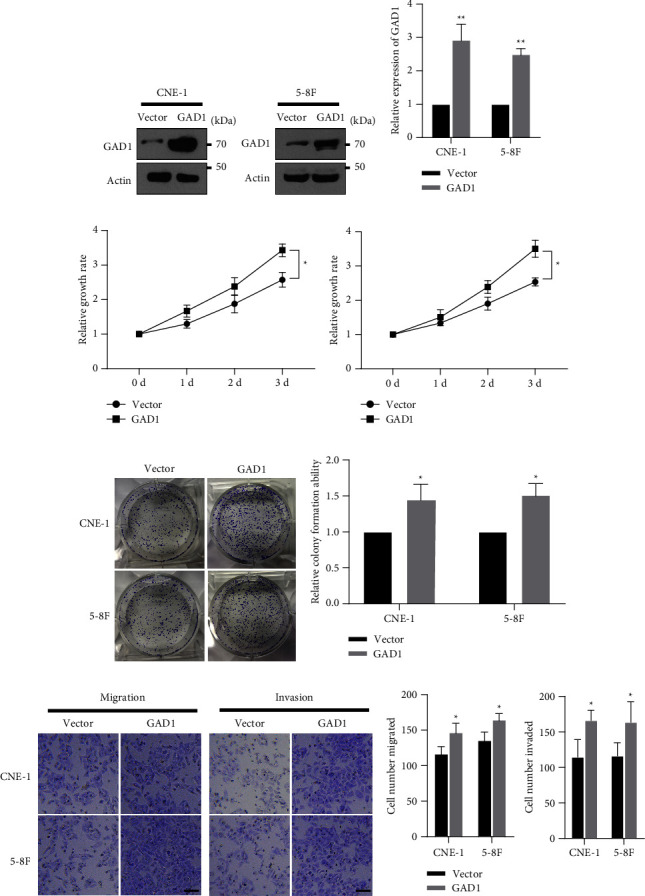
GAD1 is sufficient to promote the development of NPC cells. (a, b) GAD1 encoding plasmid was constructed and verified via transfection in NPC cells by western blotting. Then, the cells were subjected to MTT assay (c, d), colony formation assay (e, f) and Transwell assay (g, h) as in Figure 2. Scale bar, 20 *μ*m; ^*∗*^ denotes *P* < 0.05; ^*∗∗*^ denotes *P* < 0.01.

**Figure 4:**
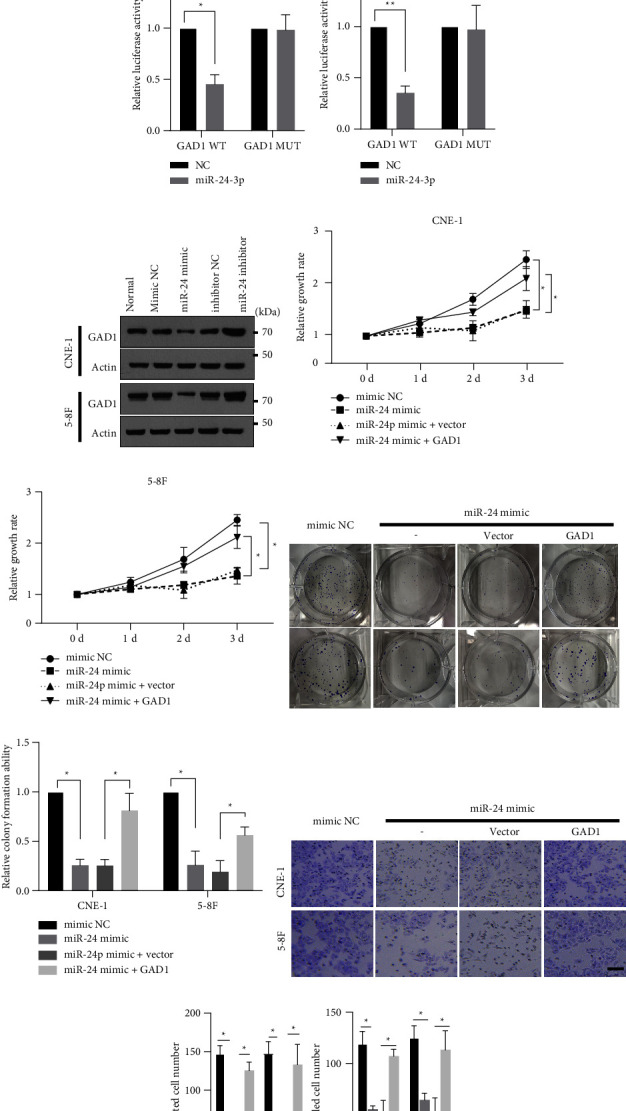
GAD1 is a target of miR-24-3p. (a) Bioinformatics analysis showed the matching site of GAD1 3′UTR to miR-24-3p, and the mutation form was constructed. (b) The homogenous of 3′UTR site of GAD1 between different species, (c, d) NPC cells were transfected with GAD1 3′UTR WT or MUT luciferase reporter, with miR-24-3p or the NC control. Then, the relative luciferase activity was determined and shown. (e) NPC cells were transfected with indicated miR-24-3p mimic or inhibitor, with mimic NC or inhibitor NC as controls. The endogenous level of GAD1 was determined by western blotting. NPC cells were transfected with miR-24-3p mimic, with or without GAD1 encoding plasmids. Then, the cells were subjected to MTT assay (f, g), colony formation assay (h, i) and Transwell assay (j, k) as in Figure 2. Scale bar, 20 *μ*m; ^*∗*^ denotes *P* < 0.05; ^*∗∗*^ denotes *P* < 0.01.

Elevated GAD1 expression in NPC tissues suggests GAD1 as a potential oncogene. To further explore its function, we designed and synthesized genetic knockdown fragments (siRNAs) against GAD1. Knockdown was first validated in two NPC cancer cell lines, CNE-1 and 5-8F, and was confirmed in both cell lines (Figures 2(a) and 2(b)). Subsequently, we determined the effects of GAD1 on NPC cell function. Transfection of siRNA fragments targeting GAD1 significantly suppressed cell differentiation in both cell lines, as indicated by an MTT assay (Figures 2(c) and 2(d)). Furthermore, transfection of GAD1-targeting siRNAs markedly suppressed the colony formation ability of both cell lines, as revealed by a colony formation assay (Figures 2(e) and 2(f)), and inhibited cell migration and invasion in both CNE-1 and 5-8F cells (Figures 2(g) and 2(h)), as demonstrated by the Transwell assay. These data suggest that GAD1 inhibition suppresses proliferation, colony formation, cell migration, and cell invasion in NPC cells. To overexpress GAD1, we cloned the GAD1 gene and transfected GAD1-encoding plasmids. GAD1 overexpression was confirmed in both cell lines (Figures 3(a) and 3(b)). GAD1 overexpression significantly promoted NPC proliferation (Figures 3(c) and 3(d)), colony formation (Figures 4(e) and 4(f)), and migration and invasion (Figures 3(g) and 3(h)) in both CNE-1 and 5-8F cells. Taken together, these data indicate that GAD1 functions as an oncogene, promoting the development of NPC cells.

### 3.3. GAD1 is the Target of miR-24-3p in Nasopharyngeal Cancer Cells

After determining the role of GAD1 in NPC, we sought to delineate the detailed regulatory mechanism for GAD1-mediated NPC development. Using online bioinformatics tools, we screened miRNAs that could target GAD1, identifying that miR-24-3p potentially targets GAD1. The potential targeting site in the GAD1 3′ UTR that matched the miR-24-3p targeting sequence is shown in Figure 4(a). The target sites of the GAD1 3′UTR were highly conserved among species (Figure 4(b)). To determine the direct targeting relationship between miR-24-3p and GAD1, we synthesized a mimic and an inhibitor of miR-24-3p and constructed reporter gene plasmids containing the wild-type (WT) or mutant (MUT) GAD1 3′UTR sequence (Figure 4(a)). For the dual-luciferase reporter gene assay, NPC cells were transfected with GAD1 reporter plasmids and the miR-24-3p mimic or empty control. Cotransfection of the miR-24-3p mimic significantly suppressed luciferase activity of the GAD1 WT reporter, but not the GAD1 MUT reporter (Figures 4(c) and 4(d)). Further, NPC cells were transfected with the miR-24-3p mimic or inhibitor, using mimic NC and inhibitor NC as controls. Transfection of the miR-24-3p mimic significantly downregulated, while transfection of the miR-24-3p inhibitor upregulated endogenous GAD1 expression in both CNE-1 and 5-8F cells (Figure 4). These findings suggested that GAD1 is a miR-24-3p target in NPC cells. Furthermore, CNE-1 and 5-8F cells were transfected with the miR-24-3p mimic with or without GAD1, and an MTT assay demonstrated that miR-24-3p suppressed NPC growth, while cotransfection with the GAD1-encoding plasmid partially restored proliferation (Figures 4(f) and 4(g)). Further, colony formation (Figures 4(h) and 4(i)), cell migration, and cell invasion (Figures 4(j) and 4(k)) were decreased by the miR-24-3p mimic, and this effect was attenuated by cotransfection with the GAD1-encoding plasmid in both CNE-1 and 5-8F cells. These data indicate that GAD1 is a direct target of miR-24-3p, and that the miR-24-3p/GAD1 axis regulates the development of NPC cells.

### 3.4. CYTOR Sponges miR-24-3p

**Figure 5:**
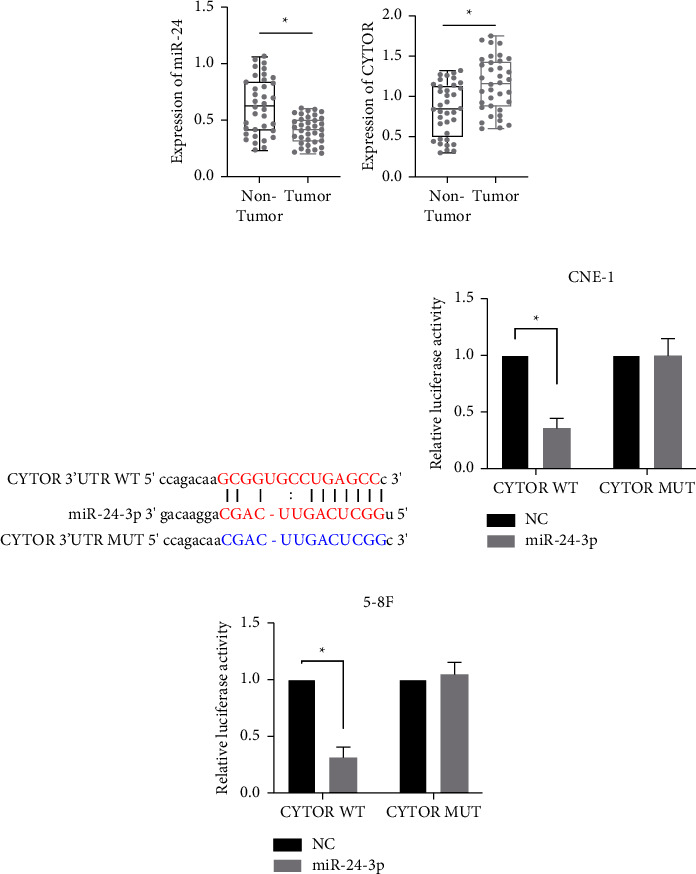
CYTOR sponges miR-24-3p. The expression levels of miR-24-3p (a) and lncRNA CYTOR (b) in our collected NPC samples were determined by RT-qPCR. (c) The bioinformatics analysis showed that CYTOR 3′UTR site matched with miR-24-3p. (d, e) NPC cells were transfected with CYTOR 3′UTR WT or MUT, together with or without miR-24-3p. Then, the luciferase activity in each group was determined and shown. ^*∗*^denotes *P* < 0.05.

We next sought to identify the upstream regulator of miR-24-3p and GAD1. LncRNA is a novel regulator that promotes NPC [31, 32]. However, the role of the lncRNA CYTOR remains unclear. We measured miR-24-3p and CYTOR expression in clinical samples of NPC and paracancerous control tissues. miR-24-3p was significantly downregulated in NPC tumor samples relative to paracancerous controls, while CYTOR was upregulated (Figure 5(a)). We conducted predictive analysis with the bioinformatics online tool starBase [33] to identify that the CYTOR 3′UTR could be matched by miR-24-3p. WT and mutant CYTOR 3′UTR sequences were constructed (Figure 5(c)). Gene reporters were cotransfected into NPC cells with either the miR-24-3p mimic or NC. miR-24-3p overexpression significantly suppressed luciferase activity of the CYTOR WT but not the CYTOR MUT reporter vector in both CNE-1 and 5-8F cells (Figures 5(d) and 5(e)). These data suggest that CYTOR could function as a sponge to suppress miR-24-3p function.

### 3.5. CYTOR/miR-24-3p Promotes Development of Nasopharyngeal Cancer Cells

**Figure 6:**
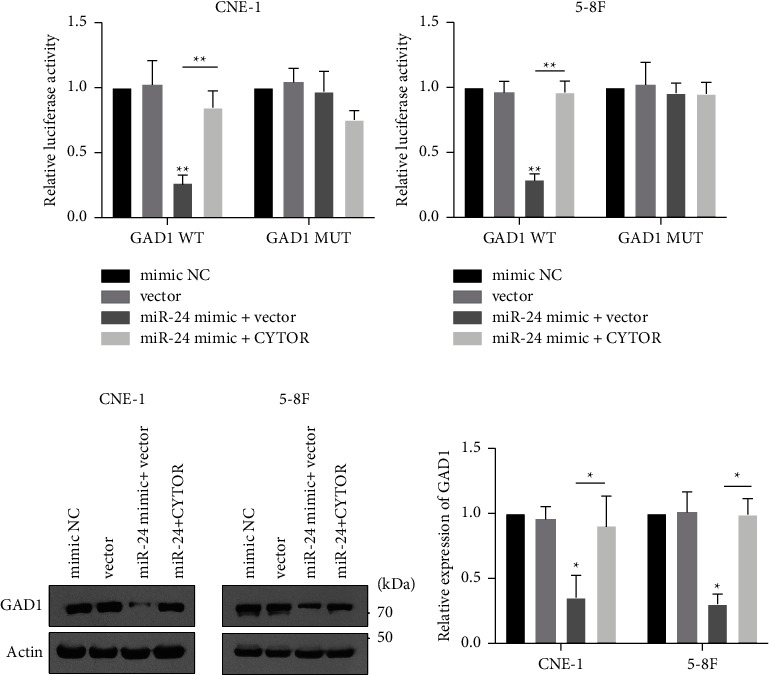
CYTOR/miR-24-3p regulates the expression of GAD1. (a, b) NPC cells were transfected with GAD1 3′UTR WT or MUT luciferase plasmid, with miR-24-3p mimic, with or without CYTOR-encoding plasmids, then the relative luciferase activity was detected. (c, d) NPC cells were treated with miR-24-3p mimic, with or without CYTOR-encoding plasmids, and the endogenous expression level of GAD1 was determined by western blotting. ^*∗*^denotes *P* < 0.05; ^*∗∗*^denotes *P* < 0.01.

Subsequently, we determined whether the CYTOR/miR-24-3p axis affected GAD1 expression. Transfection of miR-24-3p suppressed the luciferase signal of the GAD1 WT, but not the GAD1 MUT vector (Figures 6(a)). Furthermore, cotransfection of a CYTOR-encoding plasmid rescued luciferase activity in both cell lines (Figures 6(a) and 6(b)). Overexpression of the miR-24-3p mimic significantly downregulated endogenous GAD1, and cotransfection of a CYTOR-encoding plasmid abolished this inhibitory effect (Figures 6(c) and 6(d)). These data indicate that the CYTOR/miR-24-3p axis modulates GAD1 expression.

**Figure 7:**
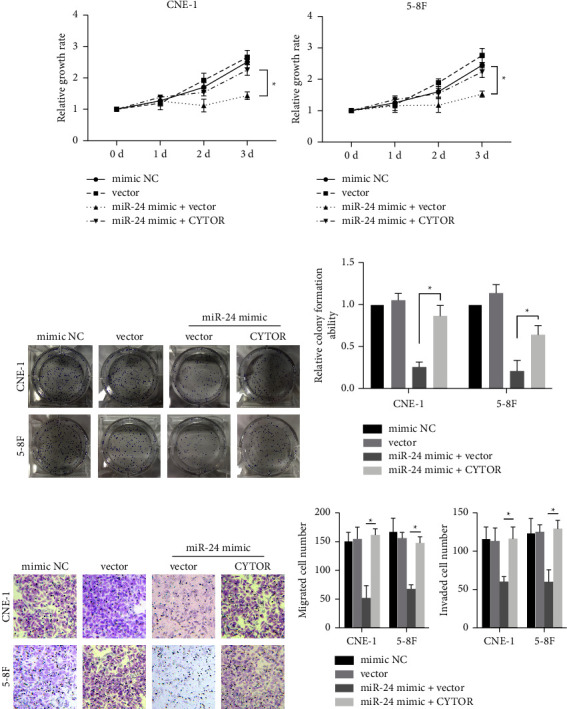
CYTOR/miR-24-3p/GAD1 axis regulates the development of NPC cells. NPC cells were treated as in Figures 6(c) and 6(d), and then the cells were subjected to MTT assay (a, b), colony formation assay (c, d), and Transwell assay (e, f). Scale bar, 20 *μ*m; ^*∗*^denotes *P* < 0.05.

Finally, we determined if CYTOR/miR-24-3p affected cell function via modulation of GAD1. Both NPC cell lines were transfected with miR-24-3p mimic fragments, with or without the CYTOR-encoding plasmid. Transfection of miR-24-3p significantly suppressed NPC cell proliferation, which was rescued by cotransfection of CYTOR (Figures 7(a) and 7(b)). Colony formation ability (Figures 7(c) and 7(d)) and cell migration and invasion ability (Figures 7(e) and 7(f)) were decreased by miR-24-3p, and this effect was counteracted by overexpression of CYTOR. These data indicate that the CYTOR/miR-24-3p axis regulates NPC cell development via modulation of GAD1 expression.

## 4. Discussion

In the present study, we demonstrated that GAD1 was upregulated in NPC tissues and functioned as an oncogene in the context of NPC. Genetic GAD1 knockdown significantly suppressed, while GAD1 overexpression promoted the growth, colony formation, migration, and invasion of NPC cells. Bioinformatics analyses and luciferase activity assays demonstrated that GAD1 was a miR-24-3p target gene. The miR-24-3p/GAD1 axis regulated the proliferation, colony formation, migration, and invasion of NPC cells. Furthermore, miR-24-3p was downregulated and the lncRNA CYTOR was upregulated in NPC tissues. CYTOR functioned as a sponge to suppress miR-24-3p activity. Thus, CYTOR regulates GAD1 luciferase activity and expression by modulating miR-24-3p. Taken together, these findings demonstrated that CYTOR/miR-24-3p/GAD1 is a critical axis for NPC development and pathogenesis.

GAD1 and GABA were first identified in neural systems and play critical roles in many neurological diseases, including Parkinson's disease, bipolar disorder, and schizophrenia [15, 34]. However, recent studies have identified that GAD1 and GABA play active roles in non-neural systems, especially in promoting cancers, including oral, breast, gastric, prostate, and colorectal cancers [8, 10, 15]. GAD1 is activated by DNA methylation in cancer cells to increase its expression, which is pivotal for the development of mucinous colon cancer [15, 35, 36]. Increased GABA and GAD1 expression are involved in prostate cancer cell migration [10, 13], and GAD1 is a specific marker for prostate cancer tissues because GAD1 expression is positively correlated with a higher Gleason score and poorer prognosis [12, 37]. GAD1 plays a crucial role and has been identified as a potential target for oral squamous cell carcinoma, due to its interaction with the Wnt/*β*-catenin/MMP7 pathway [8]. Furthermore, GAD1 influences GABA metabolism via the glutamine-glutamate/GABA cycle [38], which is important for hepatocellular carcinoma [39]. A recent study reported that GAD1 is overexpressed in NPC and is closely associated with the AJCC stage, and thus could be used as a prognosticator of poor outcomes in NPC [40]. However, the function of GAD1 in NPCs remains to be explored. In the present study, we identified that GAD1 was upregulated in NPC tissues and constructed GAD1 overexpression plasmids and siRNA fragments to elucidate the effect of GAD1 on the development of NPC cells. Furthermore, the upstream miR-24-3p/CYTOR regulator targeted GAD1 during NPC development. Further in vivo studies will be imminently conducted to elucidate the in vivo role of GAD1 and the impact of the CYTOR/miR-24-3p/GAD1 axis.

Noncoding RNAs play an important role in the occurrence, development, and malignant progression of human tumors, particularly miRNAs, lncRNAs, and circular RNAs. miRNA is a type of noncoding RNA that typically contains 20 to 25 nucleotides and binds the 3′- or 5′-UTR regions of target gene mRNA, decreasing the translation and potentially the stability of mRNA [41]. miR-24-3p promotes tumor proliferation, growth, and metastasis in the human lung, bladder, colorectal adenocarcinoma, hepatocellular carcinoma, and other tumors [42–47]. In lung cancer, miR-24-3p is upregulated and targets SOX7 to promote cancer cell development in vitro and in vivo [42]. In bladder cancer, miR-24-3p is also upregulated and targets the DEDD protein to regulate proliferation, apoptosis, migration, invasion, and autophagy [43]. In colorectal cancer (CRC), elevated expression of miR-24-3p promotes tumor growth and is closely correlated with poor disease-free survival and overall survival in colorectal adenocarcinoma patients, indicating its potential value as a prognosticator [44, 47]. In hepatocellular carcinoma (HCC), miR-24-3p is significantly upregulated and decreases metallothionein 1M expression to promote the initiation and progression of HCC [46]. The lncRNA CASC2 inhibits cell viability and induces apoptosis by targeting miR-24-3p, suppressing HCC [45]. However, miR-24-3p also has a tumor suppressive effect in colon cancer, lacrimal gland adenoid cystic carcinoma, non-small cell lung cancer, and other malignancies [48–50]. miR-24 and miR-101 are downregulated in gastric cancer [50], and Gao et al. reported that miR-24-3p is reduced in CRC samples from patients that underwent radical resection and is associated with CRC cell proliferation, migration, and invasion [48]. Pan et al. reported that decreased miR-24-3p expression favors small-cell lung cancer cells for VP16-DDP treatment by modulating its downstream target ATG4A protein during autophagy [49].

The role of miR-24-3p in human malignant tumors is therefore controversial and likely to be tissue-specific. In NPCs, the role of miR-24-3p is incompletely understood. Ye et al. reported that miR-24-3p levels are increased in exosomes from patient sera or NPC cells [51, 52], and exosomal miR-24-3p impedes T-cell function by targeting FGF11 in NPC [20]. However, in the present study, we demonstrated that miR-24-3p was downregulated in NPC tissues, unlike previous reports. We demonstrated that miR-24-3p was downregulated by the lncRNA CYTOR to promote the development of NPC cells. We did not measure circulating or exosomal miR-24-3p levels in NPC patients. To better elucidate the role of miR-24-3p, larger sample sizes should be analyzed, and the distribution of miR-24-3p expression in human organs should be characterized.

CYTOR (synonymous with C2orf59, LINC00152, and NCRNA00152) is a long noncoding RNA that is overexpressed in tumor cells and promotes cell proliferation and the epithelial-mesenchymal transition [53]. CYTOR is abnormally expressed in many tumors. Want et al. reported that CYTOR expression is positively correlated with BCL6 protein levels in ovarian cancer tissues and cell lines, in which CYTOR promotes polyubiquitination of BCL6 to support carcinogenesis [54]. CYTOR downregulation inhibits the invasiveness of cancer cells in vitro and in vivo by interacting with miR-139-5p [55–57]. In CRC, CYTOR functions as a sponge to suppress the expression and function of miRNA [58], and CYTOR inhibition upregulates miR-215, which targets CKD13 to suppress the development of liver cancer [59]. Also, lncRNAs play important roles in the development of NPCs [17]. LncRNA CYTOR regulates mitochondrial metabolism and glycolysis of oral cancer cells via HNRNPC-mediated ZEB1 stabilization in oral squamous cell carcinoma [60], and the level of CYTOR could be upregulated by forkhead box D1 to promote EMT and chemoresistance in oral squamous cell carcinoma [61]. In NPC, CYTOR titrates miR-613 to regulate ANXA2 to promote cell invasion and metastasis [21]. In the present study, we identified that CYTOR was upregulated in NPC tissues. Further, CYTOR acted as a sponge to suppress the expression of miR-24-3p and increase GAD1 levels. The CYTOR/miR-24-3p/GAD1 axis contributes to the proliferation, colony formation, migration, and invasion of NPC cells.

In summary, the present study identified the upregulation of GAD1 in NPC tissues and public databases of NPC arrays. CYTOR and GAD1 are oncogenes that promote the development of NPC cells. GAD1 is a miR-24-3p target, and CYTOR suppresses the expression of miR-24-3p to increase GAD1 levels. These data identify a novel mechanism by which the CYTOR/miR-24-3p/GAD1 axis regulates NPC development, which could provide new insights into the identification, treatment, and prognostication of NPC.

## Figures and Tables

**Table 1:** Primers used for qRT-PCR.

Gene	Fwd primer 5′-3′	Rev primer 5′-3′
miR-24-3p	AACACACCTATTCAAGGATTCA	mRQ 3′primer (clontech)
CYTOR	AGAATGAAGGCTGAGGTGTG	CAGCGACCATCCAGTCATTTA
U6	CTCGCTTCGGCAGCACA	AACGCTTCACGAATTTGCGT
Actin	TCACCCACACTGTGCCCATCTACGA	GGATGCCACAGGATTCCATACCCA

## Data Availability

The datasets used during the present study are available from the corresponding author upon reasonable request.
